# Intrahepatic cholangiocarcinoma – influence of resection margin and tumor distance to the liver capsule on survival

**DOI:** 10.1186/s12893-020-00718-7

**Published:** 2020-04-06

**Authors:** Fabian Bartsch, Janine Baumgart, Maria Hoppe-Lotichius, Beate K. Straub, Stefan Heinrich, Hauke Lang

**Affiliations:** 1 Department of General, Visceral and Transplant Surgery, University Medical Center of the Johannes Gutenberg-University Mainz, Langenbeckstraße 1, 55131 Mainz, Germany; 2 Department of Pathology, University Medical Center of the Johannes Gutenberg-University Mainz, Mainz, Germany

**Keywords:** Intrahepatic cholangiocarcinoma, Cholangiocarcinoma, Resection margin, Tumor distance, Liver capsule, Overall survival, Recurrence

## Abstract

**Background:**

Intrahepatic cholangiocarcinoma (ICC) is often diagnosed in advanced stage. Aim of this study was to analyse the influence of resection margins and tumor distance to the liver capsule on survival and recurrence in a single center with a high number of extended resections.

**Methods:**

From January 2008 to June 2018 data of all patients with ICC were collected and further analysed with Kaplan Meier Model, Cox regression or Chi^2^ test for categorical data.

**Results:**

Out of 210 included patients 150 underwent curative intended resection (71.4%). Most patients required extended resections (*n* = 77; 51.3%). R0-resection was achieved in 131 patients (87.3%) with minimal distances to the resection margin > 1 cm in 22, 0.5-1 cm in 11, 0.1–0.5 cm in 49 patients, and <  0.1 cm in 49 patients. Overall survival (OS) for margins > 0.5 cm compared to 0.5–0.1 cm or R1 was better, but without reaching significance. All three groups had significantly better OS compared to the irresectable group. Recurrence-free survival (RFS) was also better in patients with a margin > 0.5 cm than in the < 0.5–0.1 cm or the R1-group, but even without reaching significance. Different distance to the liver capsule significantly affected OS, but not RFS.

**Conclusions:**

Wide resection margins (> 0.5 cm) should be targeted but did not show significantly better OS or RFS in a cohort with a high percentage of extended resections (> 50%). Wide margins, narrow margins and even R1 resections showed a significant benefit over the irresectable group. Therefore, extended resections should be performed, even if only narrow margins can be achieved.

## Background

Intrahepatic cholangiocarcinoma (ICC) is a rare tumor and the least frequent of all bile duct cancers. Its incidence especially in the western countries is rising in the last decades [1–3]. Because of its rarity, only few survival analyses exist in the literature identifying several risk factors for poor prognosis such as tumor size, multifocality, positive lymph nodes, metastases, vascular infiltration, incomplete resection or additional liver disease [4–6].

Currently, complete tumor resection is considered the only chance for cure. The role of positive or negative resection margin is extensively investigated in other primary or secondary liver malignancies such as colorectal liver metastases (CRLM) or hepatocellular carcinoma (HCC), but still controversial for ICC. Even between CRLM and HCC, the importance of minimal resection margin differs. While in CRLM the impact of the resection margin on long-term outcome is associated with the response to chemotherapy [7, 8], larger resection margins are associated with better survival for HCC patients [9]. Similarly, the extent of the resection margin has been suggested also to be important for ICC in single [10] or multicentre studies [11, 12]. However, the importance of the distance to the liver capsule has not been analysed for intrahepatic cholangiocarcinoma at all.

The aim of this current study was to investigate the influence of the distances to the resection margin as well as to the liver capsule on recurrence-free and overall survival as well as the pattern of recurrence in a single center.

## Methods

All patients undergoing exploration for liver resection were collected in a prospective institutional database. Only patients who underwent explorative laparotomy for ICC between January 2008 and June 2018 qualified for this analysis. Patients with mixed ICC/HCC tumors were excluded from this analysis. Tumors with origin in the perihilar bile ducts recognizable by biliary intraepithelial neoplasia were excluded from the study. Data of eligible patients were transferred to a SPSS 23 (SPSS Inc. Released 2014, IBM SPSS Statistics for Windows, Version 23.0, IBM Armonk, NY, USA: IBM Corp.) database for further analysis.

All patients signed informed consent that data and follow-up will be collected anonymously and is potentially used for scientific analysis. Regarding to the regulations of the federal state law (state hospital law §36 & §37) and the independent ethics committee of Rheinland-Palatinate, no ethical approval was necessary for this study.

### Staging procedures – surgery – follow up

Preoperative staging was based on high resolution computed tomography (CT) and/or magnetic resonance imaging (MRI) of good quality. If not previously performed elsewhere, we routinely performed colonoscopy and gastroscopy to exclude a primary gastrointestinal tumor. A preoperative biopsy does not belong to our routine work-up, but some patients were referred after histological proof of ICC.

All procedures were performed by the surgical HPB team. Surgery for ICC routinely includes a standard hilar lymphadenectomy. Follow-up was performed every 3 months for at least 2 years and based on CT-scan or MRI 3 months after surgery, and every 6 months thereafter. In between patients underwent ultrasound. After 2 years we recommended the patients to continue CT or MRI imaging every 6 months, but offered continuation of ultrasound as well. Whenever follow-up was performed outside our center due to the distance from their homes, we contacted the referring physician for all necessary information.

### Data analysis

The surgical procedures, morbidity, mortality, histological results, recurrence-free and overall survival were analysed. Major and minor resections were classified according to the Brisbane-classification [13]: extended resections were defined as ≥5 resected segments and included mesohepatectomy, associating liver partition and portal vein ligation for staged hepatectomy (ALPPS) and all resections requiring the resection of surrounding organs or vessels.

The UICC 8th edition was used for disease staging [14]. Surgical morbidity was classified according to Clavien-Dindo [15], and mortality includes all in-hospital deaths as well as those occurring within 30- and 90-days from surgery.

### Resection margins and tumor-relation to the liver capsule

The extent of the resection margin was grouped: > 2 cm, 1-2 cm, 0.5-1 cm, 0.1–0.5 cm, < 0.1 cm or R1.

In addition, the distance of the tumor to the liver capsule was quantified as “distant” (> 0.5 cm), “close/infiltration (< 0.5 cm)” or perforation. The distance of 0.5 cm was chosen because of a definition through our department of pathology that no detailed distance was given, if the distance exceeded 0.5 cm. Additionally, a group of patients showed centrally located ICC with dissemination/infiltration of the hepatic hilum. Because of the periductal dissemination and growth out of the liver parenchyma we defined these patients as own subgroup. The preoperative imaging, clinical and intraoperative features of these tumors (tumor diameter > 3 cm, located in second or third order bile ducts, imaging like a centrally located ICC) argued for them to be ICC involving the liver hilum, like described before [16, 17].

### Statistical analysis

Only patients with complete data-sets were included in the statistical analyses. Statistics for categorial data was performed with the χ^2^-Test. The Kaplan Meier model was used for survival analyses, and the log rang test was used for the comparison of factors. Perioperative deaths were excluded from survival analyses. Multivariate analysis was performed using the Cox regression model.

## Results

Of 210 patients, who all underwent exploration for curative intended resection, 150 underwent liver resection with curative intent (71.4%). Reasons for irresectability (*n* = 60) were peritoneal carcinomatosis (*n* = 23), multifocal tumor dissemination (*n* = 15), locally advanced infiltration (*n* = 11) or cirrhosis/small for size liver remnant/poor quality of parenchyma (*n* = 11).

Gender was nearly equally distributed in the resection group (♀ *n* = 73; ♂ *n* = 77) with a median age of 64.2 years (IQR 56–73.7; range 32.3–84.4). The median BMI was 26.1 (IQR 23.8–29.3) while most patients were ASA II (*n* = 62) or III (*n* = 83) [ASA I *n* = 2, IV *n* = 3]. The majority of patients (*n* = 106) required major or even extended liver resections, and 131 patients underwent an R0 resection (87.3%). The majority of tumors was locally advanced (Table 1).

**Table 1:** Surgical procedures and histological outcome

	All	R0				R1
**Resection Margins**		**> 1 cm**	**0.5–1 cm**	**0.1–0.5 cm**	**< 0.1 cm**	**pos.**
	***n*** **= 210**	***n*** **= 22**	***n*** **= 11**	***n*** **= 49**	***n*** **= 49**	***n*** **= 19**
**Primary resection**	***n*** **= 150**					
Right trisectionectomy	**26**	1	1	8	12	4
Left trisectionectomy	**22**	3	–	5	8	6
Right hepatectomy	**25**	4	1	9	9	2
Left hepatectomy	**19**	5	2	4	7	1
Mesohepatectomy ^a^	**7**	–	–	4	2	1
ALPPS	**6**	1	1	3	1	–
Monosegmentectomy	**9**	2	1	5	1	–
Bisgementectomy	**25**	3	5	9	5	3
Resection of three liver sg.	**9**	2	–	2	4	1
Atypic / wedge resection	**2**	1	–	–	–	1
**irresectable**	***n*** **= 60**	–	–	–	–	–
**Histology (TNM 8th Ed.)**						
**T status**						
T1a	**23**	4	5	8	5	1
T1b	**32**	6	–	15	7	4
T2	**59**	7	4	15	24	9
T3	**14**	1	1	6	5	1
T4	**22**	4	1	5	8	4
**N status**						
N0	**90**	19	7	26	26	12
N1	**43**	3	2	14	18	5
N2	**1**	–	–	–	1	–
Nx	**19**	–	2	9	4	2
**R status**						
R0	**131**	–	–	–	–	–
*> 2 cm*	*5*	5	–	–	*–*	–
*1–2 cm*	*17*	17	–	*–*	–	–
*0.5–1 cm*	*11*	–	*11*	–	–	–
*0.1–0.5 cm*	*49*	*–*	–	49	–	–
*< 0.1 cm*	*49*	*–*	–	–	49	–
R1	**19**	–	–	–	–	*19*
**Tumor relation to capsule**						
Distant	**50**	7	3	19	16	5
Close/Infiltration	**78**	11	7	26	25	9
Periductal dissemination	**11**	1	1	2	5	2
Perforation	**11**	3	–	2	3	3
**Grading**						
G1	**3**	1	–	1	1	–
G2	**92**	15	9	33	28	7
G3	**43**	3	2	13	16	9
G4	**1**	–	–	–	1	–
Preoperative chemotherapy	**11**	3	–	2	3	2
**UICC stage (TNM 8th Ed.)**^b^						
IA	**15**	3	4	4	4	–
IB	**19**	6	–	6	4	3
II	**36**	6	2	10	13	5
IIIA	**8**	1	–	3	3	1
IIIB	**47**	4	2	16	18	7
IV	**8**	2	1	1	3	1

^a^ ≥ three central segments; ^b^ 17 patients with Nx do not have a UICC stage

### Visceral and vascular extensions

In total, liver resection was extended in 102 cases by visceral and/or vascular resections in 61 patients. Neither the infiltration (*p* = 0.286) nor the resection of surrounding viscera (*p* = 0.26) were prognostic for the status of the resection margin (R0/R1). Also, the resection of surrounding vessels (e.g. portal vein, vena cava, *p* = 0.077) and the infiltration of such vessels (*p* = 0.389) had no influence on the margin status (R0 or R1).

#### Morbidity and mortality

Complications occurred in 69 patients (46%), of which 17 patients had minor complications (Dindo I + II). Treatment relevant complications (Dindo IIIa – IVb) occurred in 39 patients, and 13 patients (8.7%) died in the postoperative course due to sepsis (*n* = 3), liver (*n* = 4) or multi-organ (*n* = 6) failure. Twelve of these deaths occurred within 30 days, while one patient died within 90 days.

#### Factors associated with small resection margins and the distance to the liver capsule

A R0 resection was achieved in 131 patients (87.3%), and 19 resections were classified as R1, but none as R2. Subgroups according to established risk factors were analysed regarding their relevance for achieving a sufficient resection margin. Since the median resection margin reached 0.1 cm, patients were grouped for ≤0.1 cm vs. > 0.1 cm, and the association of the established risk factors were tested in cross tabulation: gender (*p* = 0.009), major resection (*p* = 0.004), extended resection (*p* = 0.029), vascular extension and reconstruction (*p* = 0.005), vascular infiltration (*p* = 0.047), tumor grading (G1 + 2 vs G3 + 4; *p* = 0.024) and T stage (*p* = 0.027) were significantly associated with resection margins.

The distance of the tumor to the liver capsule was analysed in the same way. Groups for cross tabulation were distant (*n* = 50), close/infiltration (*n* = 78) and perforation of the capsule (*n* = 11) in combination with the periductal dissemination growth type (*n* = 11). Significance was reached for extended resection (*p* <  0.001), T stage (*p* < 0.001), visceral extension (*p* < 0.001), visceral infiltration (*p* < 0.001), multifocality (*p* = 0.001) and UICC stage (UICC I + II vs. UICC III + IV; *p* = 0.002).

#### Tumor recurrence

Ninety-six patients (64%) developed a tumor recurrence within a median follow-up of 62.5 months. Most recurrences developed within the liver only (*n* = 42, 43.8%), while about a third of recurrences each were detected within and outside the liver (*n* = 29, 30.2%) or only outside the liver (*n* = 25, 26%). Different resection margins had no significant influence on the location of tumor recurrence (*p* = 0.354), neither had tumor distance to the liver capsule (*p* = 0.072).

The majority of recurrences (*n* = 60) was treated by palliative chemotherapy, and 12 patients only received best supportive care. Eleven patients qualified for repeat liver (*n* = 9) or extrahepatic (*n* = 2) resection, and another 6 patients underwent tumor ablation. In addition, four patients were treated by trans-arterial chemoembolization (TACE) and selective internal radiotherapy (SIRT), stereotactic irradiation or palliative surgery, once each, due to a limited intrahepatic but unresectable recurrence. Therapy of recurrence did not differ significantly regarding resection margins (*p* = 0.404) or distance to the liver capsule (*p* = 0.874).

#### Survival analysis

##### Influence of the extent of the resection margins and tumor distance to the liver capsule on overall survival

The median overall survival (OS) in an intention to treat analysis was 21.6 months with consecutive 1-, 3- and 5-year OS rates of 72, 29 and 16%, respectively. After excluding perioperative deaths, the median OS was 23.6 months with consecutive 1-, 3- and 5-year OS rates of 79, 32 and 17%, respectively.

**Fig. 1:**
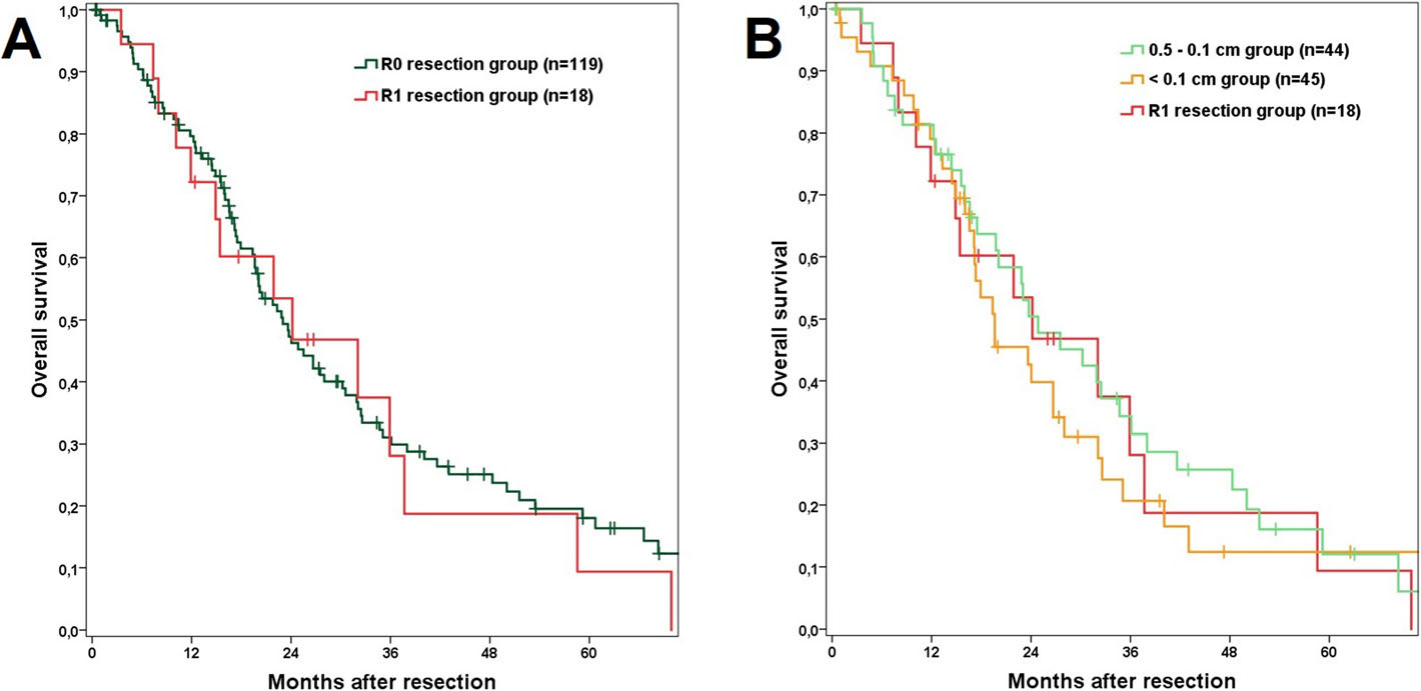
**a** Kaplan Meier curve of the R0 and R1 resection groups comparing overall survival. *p* = 0.655; perioperative deaths were excluded. **b** Kaplan Meier curve comparing overall survival of the resection margin subgroups R1, < 0.1 cm and 0.1–0.5 cm with a comparable outcome. *p* = 0.732; perioperative deaths were excluded

**Fig. 2:**
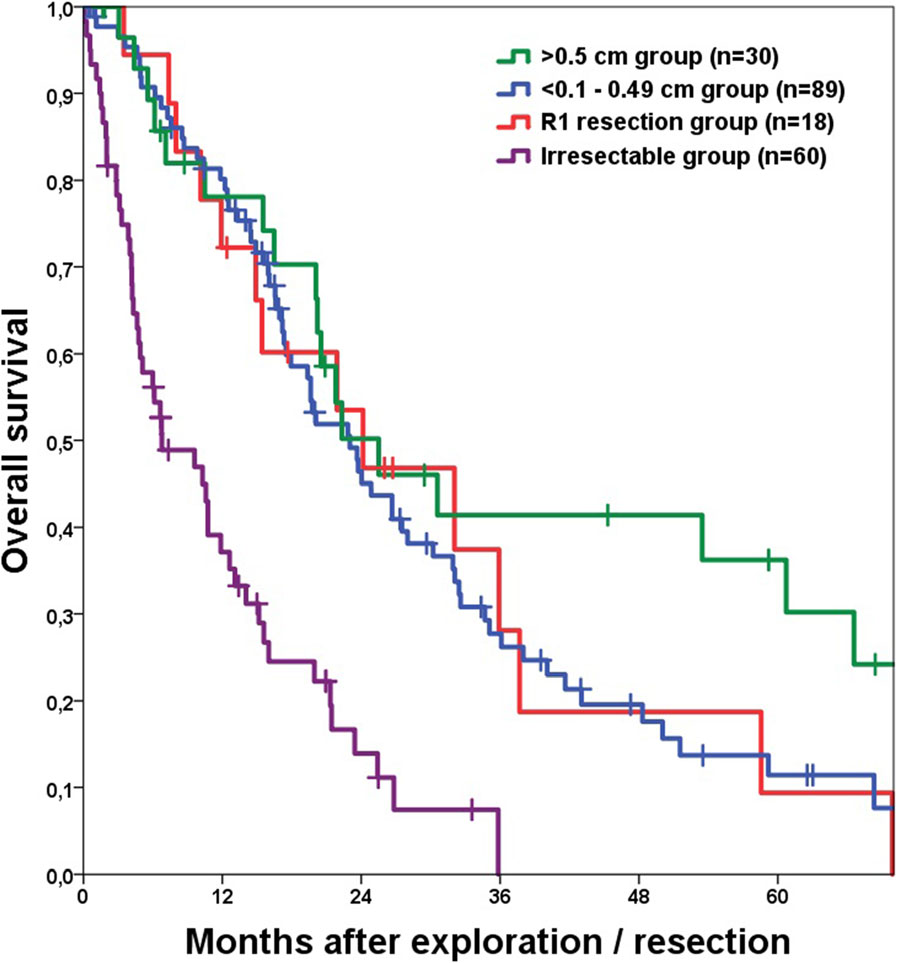
Kaplan Meier curve comparing overall survival of the > 0.5 cm, 0.5–0.1 cm, R1 resection and Irresectable group. Between the > 0.5 cm, 0.5–0.1 cm and R1 groups no significant difference in overall survival could be shown. But all of these groups had a favourable outcome compared to the Irresectable group (*p* < 0.001 for > 0.5 cm and 0.5–0.1 cm; *p* = 0.001 for R1); perioperative deaths were excluded

A comparison of R0 versus R1 resections showed no significant survival difference (*p* = 0.655; Fig. 1a, Table 2). Also, the OS rates of the R1, < 0.1 cm and 0.1–0.5 cm groups were comparable (*p* = 0.732; Fig. 1b). A margin > 0.5 cm was associated with a longer OS, although the difference did not reach significance (*p* = 0.087; Fig. 2). All resected patients had a significantly better OS than patients with irresectable disease (*p* < 0.001; Fig. 2).

**Table 2:** Overall and recurrence-free survival of the resection margin groups

	n	Median	1-year	3-year	5-year	*p*-value
		months	%	%	%	
**Overall survival**						
*Resection group*	*137*	*23.6*	*79*	*32*	*17*	
> 0.5 cm	30	24.5	79	42	36	< 0.001
0.49–0.1 cm	44	25	81	35	12	
< 0.1 cm	45	21.8	79	22	13	
R1 resection	18	26.2	72	31	10	
*Irresectable group*	*60*	*9.6*	*37*	*4*	*–*	
**Recurrence-free survival**						
*Resection group*	*137*	*9.7*	*38*	*16*	*12*	
> 0.5 cm	30	12	50	33	27	0.166
0.49–0.1 cm	44	9.2	35	11	8	
< 0.1 cm	45	10	40	15	10	
R1 resection	18	8.3	28	–	–	

Perioperative deaths were excluded

The tumor distance to the liver capsule had a significant influence on OS (*p* = 0.033; Table 3): the distant (*p* = 0.007) and close/infiltration groups (*p* = 0.032) had a significantly better OS compared to the periductal dissemination group, while no other cross testing led to significant differences.

**Table 3:** Overall and recurrence-free survival of tumor proximity to liver capsule groups

	n	Median	1-year	3-year	5-year	*p*-value
		months	%	%	%	
**Overall survival**						
Distant	45	28	86	40	21	0.033
Close / Infiltration	73	24.1	77	32	18	
Perforation	9	20.3	76	23	–	
Periductal dissemination	10	14.5	58	–	–	
**Recurrence-free survival**						
Distant	45	10.4	43	24	20	0.142
Close / Infiltration	73	9.3	35	13	11	
Perforation	9	16.5	65	13	–	
Periductal dissemination	10	8.1	26	–	–	

Perioperative deaths were excluded

##### Influence of extent of the resection margins and tumor distance to the liver capsule on the recurrence-free survival

The median recurrence-free survival (RFS) was 9.7 months with a consecutive 1-, 3- and 5-year RFS rates of 38, 16 and 12%, respectively.

We observed a trend to a lower recurrence rate in case of a resection margin > 0.5 cm (*p* = 0.076), but the distance to the liver capsule did not reveal such a trend (*p* = 0.706).

**Fig. 3:**
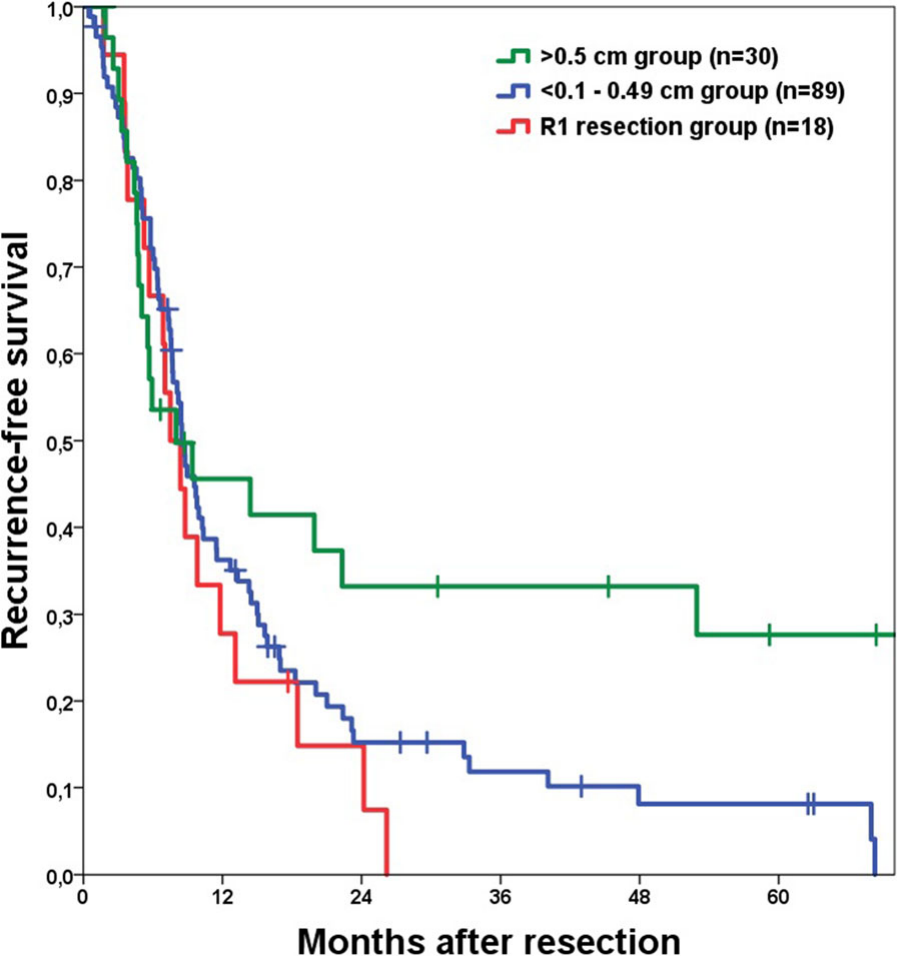
Kaplan Meier curve for recurrence-free survival of the > 0.5 cm, the 0.5–0.1 cm and R1 resection group showing no significant difference. Perioperative deaths were excluded

The most favourable outcome was observed in patient with a resection margin > 0.5 cm (Table 2), who had a significantly longer RFS than patients with a smaller resection margin (*p* = 0.040; Fig. 3).

Also, for the distance of the tumor to the liver capsule, we found a trend for a longer RFS, which however, did not reach statistical significance (*p* = 0.142, Table 3). All patients with a perforated liver capsule or a periductal dissemination recurred within 5-years from surgery (Table 3).

##### Multivariate analyses

Several parameters were tested in univariate analysis and included in multivariate analysis (Table 4). For overall survival beneath tumor distance to the liver capsule, age, major resection, tumor size (> 10 cm vs. < 10 cm) and N-stage showed to be independent predictors. For recurrence-free survival resection margin, tumor size (> 10 cm vs. < 10 cm), multifocality and M-stage were independent predictors.

**Table 4:** Univariate and multivariate analyses

		Kaplan Meier Model		Multivariate Cox regression model					
		OS	RFS	OS			RFS		
				HR	95% CI	*p*-value	HR	95% CI	*p*-value
**Resection margin**	> 0.5 cm / 0.49–0.1 cm / R1	0.230	0.111						
	> 0.5 cm / < 0.49 cm	0.087	0.052	0.754	0.428–1.326	0.326	**0.557**	**0.331–0.936**	**0.027**
	≤0.1 cm / > 0.1 cm	0.291	0.458						
**Tumor distance to liver capsule**	dist./cl. + infil./perf./pd. Infil.	**0.033**	0.136	**1.271**	**1.058–1.525**	**0.010**			
**Age**	< 65 / > 65	0.073	0.175	**1.536**	**1.026–2.300**	**0.037**			
**Gender**	Woman / Man	0.283	0.472						
**Extended resection**	yes / no	**0.009**	**0.024**	0.848	0.514–1.400	0.519	0.884	0.553–1.413	0.606
**Major resection**	yes / no	**0.045**	**0.008**	**0.573**	**0.351–0.936**	**0.026**	0.735	0.464–1.165	0.191
**Tumor size**	≤5 cm / > 5 cm	0.108	**0.008**						
	≤ 10 cm / > 10 cm	**0.014**	**0.002**	**1.609**	**1.007–2.570**	**0.047**	**1.973**	**1.272–3.061**	**0.002**
**Multifocality**	yes / no	0.262	**0.014**				**1.593**	**1.069–2.374**	**0.022**
**T-stage**	T1 + T2 / T3 + T4	0.102	0.347						
**N-stage**	N0 / N+ / NX	**0.016**	0.085	**1.258**	**1.027–1.541**	**0.026**	1.147	0.931–1.412	0.197
**V-stage**	V0 / V1 + V2	0.149	0.818						
**L-stage**	L0 / L1	0.369	0.673						
**Pn-stage**	Pn0 / Pn1	**0.027**	0.091	1.401	0.882–2.227	0.154	1.157	0.748–1.789	0.512
**M-stage**	M0 / M1	0.125	**0.002**				**2.984**	**1.347–6.612**	**0.007**
**R-stage**	R0 / R1	0.655	0.254						
**Grading**	G1 + G2 / G3 + G4	0.347	0.535						

Perioperative deaths were excluded for statistical analyses; significant parameters are bold; parameters with *p* < 0.1 were included in multivariate analyses (underlined); *OS* overall survival, *RFS* recurrence-free survival, *HR* hazard ratio, 95% CI = 95% confidence interval, dist./cl. + infil./perf./pd. Infil. = distant / close+infiltration / perforation / periductal infiltration

## Discussion

We report on a single center cohort with a high number of extended resections and vascular or visceral extensions. The aim was to demonstrate the influence of resection margins and tumor distance to the liver capsule on survival and the pattern of recurrence. Neither resection margin width nor tumor distance to the liver capsule influenced the pattern of recurrence. For resection margins we were able to show that margins > 0.5 cm had a better long-term OS and RFS, but without reaching significance in direct comparison. Nevertheless, in multivariate analysis resection margins > 0.5 cm showed to be one independent predictor for RFS. In case of tumor distance to the liver capsule a significant impact on OS could be shown, while RFS was not influenced significantly. Multivariate analysis confirmed this finding with tumor distance to the liver capsule showed to be an independent predictor for OS.

Complete resection is the goal in oncologic surgery, but especially in liver surgery, different factors like multifocality or advanced tumor growth due to late diagnosis with or without infiltration of surrounding organs or structures may lead to borderline resectability and the necessity of extended resections [6, 18–20].

In the absence of treatment alternatives, we offer liver surgery whenever a tumor appears technically resectable. Due to the extent of ICC in many cases, more than half of our patients required extended resections regarding liver volume or perihepatic structures.

Even if liver surgery and extended liver resections have evolved over the last decades, intrahepatic cholangiocarcinoma has still a bad prognosis after resection with 5-year overall survival rates between 21 and 35% [4–6, 21, 22]. We achieved a 5-year survival of 17% which is most likely explained through our aggressive attitude. In our resection group, we had a total morbidity of 46% (Clavien-Dindo I – V). Major complications (Clavien-Dindo III – V) occurred in 34.7% of cases with a mortality of 8.7%, which is comparable to the literature [4, 23, 24], especially considering the fact that extended resections were performed frequently.

Surgery remains the only chance of cure taking into account, that ablation may also lead to complete tumor clearance. Because of the late onset of symptoms ICC is often diagnosed when ablation is not feasible or possible anymore. Therefore, ablation has its role in the treatment of recurrent ICC mainly [25].

Resection margins are important and because of their prevalence more often and better analyzed in liver surgery for colorectal liver metastasis (CRLM) or hepatocellular carcinoma (HCC). While for CRLM clear margins are most important in patients who do not respond well to chemotherapy [7, 8], Zhong and colleagues showed for HCC on 1932 patients that wide surgical margins > 1 cm significantly improve survival [9]. In contrast and respect of a much smaller cohort (*n* = 130) Field and colleagues presented a comparable overall and recurrence-free survival for narrow (< 0.5 cm) and wide (> 0.5 cm) groups for HCC [26]. For ICC especially, studies with large cohorts are lacking due to its low incidence. While the impact of resection margins on survival varied in smaller cohorts [10, 27–29], papers of Yeh and colleagues (*n* = 224, single-center, analyzed period of 30 years) with a larger and especially Spolverato and colleagues (multi-center [12 centers], analyzed period 23 years) with the largest cohort (*n* = 584) were able to show a significant influence on survival [11, 30]. In two meta-analysis of Li (comparing R0 vs R1) and Tang (comparing margins > 1 cm vs. < 1 cm) and colleagues, both were able to show significant survival benefits for the R0 respectively the > 1 cm groups [31, 32]. We were able to show a survival benefit for margins > 0.5 cm, but without statistical significance. The size of our cohort is not comparable to the beforementioned cohorts, which is of course a limitation of our analysis. Nevertheless, Yeh et al. analyzed a very long time period (30 years) in a single-center and Spolverato et al. a much longer time period (23 years) of a multi-center data. Therefore, our single center cohort with an analyzed period of ten and a half years is most probably more homogenous than the above-mentioned cohorts.

In addition, we performed many extended resections with either visceral and/or vascular extensions. Visceral or vascular extension did not influence R0 or R1 resection, but with the median margin width of 0.2 cm as cut off, gender, vascular extension, T stage, major or extended hepatic resection had significant influence. Gender was nearly balanced in our cohort. Interestingly, Spolverato and colleagues reported a strong impact of gender on resection margins as well (*p* < 0.001 [11];). Further analysis of our data showed that women had significantly more frequent tumors > 7 cm (median 6.4 cm) than men (*p* = 0.039). The T stage is an expression of infiltration depth including e.g. multifocality and higher T stages are more likely to be borderline resected. The same applies for vascular extension and both together explain the need for major or extended resection. These results are according to findings of Spolverato et al. as well [11].

Tumor recurrence is common after resection of ICC and the main reason for poor long-term survival. Most often recurrence is located isolated intrahepatically with up to 60%, followed by intra- and extrahepatic (ca. 20%) or isolated extrahepatic (ca. 20%) recurrence [33, 34]. The negative influence of narrow resection margins on recurrence or recurrence-free survival is demonstrated in different studies [6, 11, 27, 28]. Accordingly, we were able to show that margins > 0.5 cm led to a significant benefit for recurrence-free survival. Data on this special topic are scarce. Until 2017, no data of prospective randomized trials were available and the adjuvant treatment of ICC after resection was not standardized. Ercolani and colleagues showed that adjuvant treatment with Gemcitabine based mono or combined treatment led to a significant benefit in overall survival [35]. Wirasorn et al. were able to show beneath the general benefit of adjuvant treatment, that the combination of Gemcitabine and Capecitabine led to the best long-term outcome [36]. We did not apply any standard adjuvant chemotherapy until the first results of the BILCAP trial got available [37]. Capecitabine is now standard adjuvant treatment after resection. Some patients with unfavorable histological results were offered an individualized consultation with our oncologists and some were included in the early ACTICCA-trial. Alternative adjuvant treatment modalities like transarterial chemoembolization (TACE), radiation alone or chemotherapy in combination with radiation might play a role in the future [38–40]. We did not consider any of these alternatives as adjuvant treatment, because prospective randomized data does not exist, at least not yet.

We performed a multivariate analysis of several clinical and histological factors known for influencing OS and RFS for ICC. For OS tumor distance to the liver capsule showed to be an independent predictor while resection margins did not. Further age, major resection, tumor size and N-stage showed to be significant. Within the literature especially multifocality, N-stage or R-stage showed to be independent predictors for OS [4, 5, 22, 41]. Regarding RFS resection margins with a cut-off of 0.5 cm showed to be significant while tumor distance to the liver capsule did not. Further independent predictors for RFS were tumor size, multifocality and M-stage. Resection margins do not influence OS but RFS in our cohort with a high percentage of extended resections. This may be explained by the importance of tumor resection for OS, while recurrence is obviously and understandable depending on wider resection margins.

To the best of our knowledge the influence of tumor distance to the liver capsule on overall and disease-free survival has not been analysed yet. We were able to show a significant benefit for overall survival, especially for the distant and close/infiltration groups, but no influence on recurrence-free survival. In addition to distant, close/infiltration and perforation groups we specially classified a periductal dissemination type. We observed that some ICC deriving from centrally located bile ducts spread into the liver hilum without perforating the liver capsule. This special type is most likely comparable to the hilar type ICC described by Zhang and colleagues [16]. In their study the hilar type ICC had significant worse outcome compared to peripheral ICC (and perihilar cholangiocarcinoma), which matches our findings. All patients of the periductal dissemination group had a survival less than 3 years. One may argue that these tumors are perihilar cholangiocarcinoma. But as mentioned in the methods section, imaging, clinical and intraoperative features argued for these tumors to be ICC involving the liver hilum, as described by Zhang and Murakami et al. [16, 17]. Furthermore, the median diameter of these tumors was 5.7 cm (IQR 4.1–9.3; Mean 6.4 cm), which is highly uncommon, especially for resectable perihilar cholangiocarcinoma.

Complete tumor clearance with vascular and visceral extensions may explain why tumor distance to the liver capsule did not predict RFS in multivariate analysis. This is different in case of OS, imaginable especially due to the worse tumor biology in case of tumors with perforation of the liver capsule or the periductal dissemination type.

## Conclusion

In conclusion, resection margins > 0.5 cm, < 0.1–0.5 cm or R1 resections showed no significant difference for overall and recurrence-free survival. All of these groups had a clear and significant benefit over irresectable tumors. Therefore exploration and if necessary extended resection should be considered, even if only narrow margins can be achieved. Tumor distance to the liver capsule showed an influence on overall and recurrence-free survival with disadvantages especially for the perforation or periductal dissemination groups.

## Data Availability

The datasets used and analyzed during the current study are available from the corresponding author on reasonable request.

## Abbreviations

ALPPS: Advanced liver partition and portal vein ligation for staged hepatectomy
ASA: American Society of Anaesthesiologists
CRLM: Colorectal liver metastasis
CT: Computed tomography
HCC: Hepatocellular carcinoma
ICC: Intrahepatic cholangiocarcinoma
IQR: Interquartile range
MRI: Magnetic resonance imaging
OS: Overall survival
RFS: Recurrence-free survival
SIRT: Selective internal radiotherapy
TACE: Trans-arterial chemoembolization
